# A Complete Guide to Extraction Methods of Microplastics from Complex Environmental Matrices

**DOI:** 10.3390/molecules28155710

**Published:** 2023-07-28

**Authors:** Monika Rani, Serena Ducoli, Laura Eleonora Depero, Miljana Prica, Aleksandra Tubić, Zahida Ademovic, Liam Morrison, Stefania Federici

**Affiliations:** 1 Department of Mechanical and Industrial Engineering, University of Brescia and INSTM Research Unit of Brescia, 25123 Brescia, Italyserena.ducoli@unibs.it (S.D.); laura.depero@unibs.it (L.E.D.); 2 Faculty of Technical Sciences, University of Novi Sad, 21000 Novi Sad, Serbia; miljana@uns.ac.rs; 3 Faculty of Sciences, University of Novi Sad, 21000 Novi Sad, Serbia; aleksandra.tubic@dh.uns.ac.rs; 4 Faculty of Forestry, University of Sarajevo, 71000 Sarajevo, Bosnia and Herzegovina; 5 Earth and Ocean Sciences, School of Natural Sciences and Ryan Institute, University of Galway, H91TK33 Galway, Ireland

**Keywords:** microplastics, extraction methods, complex environmental matrices, emerging contaminants

## Abstract

Sustainable development is a big global challenge for the 21st century. In recent years, a class of emerging contaminants known as microplastics (MPs) has been identified as a significant pollutant with the potential to harm ecosystems. These small plastic particles have been found in every compartment of the planet, with aquatic habitats serving as the ultimate sink. The challenge to extract MPs from different environmental matrices is a tangible and imperative issue. One of the primary specialties of research in environmental chemistry is the development of simple, rapid, low-cost, sensitive, and selective analytical methods for the extraction and identification of MPs in the environment. The present review describes the developments in MP extraction methods from complex environmental matrices. All existing methodologies (new, old, and proof-of-concept) are discussed and evaluated for their potential usefulness to extract MPs from various biotic and abiotic matrices for the sake of progress and innovation. This study concludes by addressing the current challenges and outlining future research objectives aimed at combating MP pollution. Additionally, a set of recommendations is provided to assist researchers in selecting appropriate analytical techniques for obtaining accurate results. To facilitate this process, a proposed roadmap for MP extraction is presented, considering the specific environmental compartments under investigation. By following this roadmap, researchers can enhance their understanding of MP pollution and contribute to effective mitigation strategies.

## 1. Introduction

With science and innovation present in the DNA of plastic, the plastic industry has become intrinsic to the global economy. Accompanying its unparalleled features, such as its low-cost production and availability, durability, and high strength-to-weight ratio, plastic has become an indispensable product in our day-to-day life, allowing us to meet a myriad of aesthetic and functional demands. According to a report published in *Plastics Product Global Manufacturing Global Market 2017*, the plastic industry is growing by 3% every year [1]. However, the disposal of post-consumer plastic waste has been a global concern from the beginning of the plastic era. Mismanaged plastic waste is discarded in water bodies and landfills, where it is subjected to either physical, chemical, or biological degradation processes, which transform it into smaller fragments called microplastics (MPs).

The ISO defines MPs as “any water-insoluble plastic particle with its longest dimension between 1 µm and 1 mm” [2]. They are further classified as primary MPs when produced intentionally in the form of pellets, granules, fibers, or powders for use in personal care products and are called secondary MPs when fragmented from larger plastics (macroplastics or plastic debris) due to external forces (UV degradation, wind, water current, and washing of clothes). In addition, based on dimension, ISO classifies large MPs as any water-insoluble solid particle with dimensions between 1 mm and 5 mm. For many years following the publication of the first report on MPs in sediments in 2004 [3], the study of MPs was predominately confined to the marine environment, focusing on their occurrence in the oceans and along beaches, with very few studies reporting the interaction with aquatic organisms. Subsequently, researchers began to examine other environmental compartments and found that MPs were widely present, ranging from Arctic Sea ice to the hadal sediments of the Mariana Trench [4], from drinking water to wastewater, from soil to sewage sludge, and in many species, including humans. As plastics do not behave similarly in different matrices, it is fundamental to trace their origin, path, and fate for the understanding of the mitigation strategies and their effect on organisms. MPs impact animals in two ways: physically via ingestion or entanglement; and chemically due to the presence of additives, adsorbed organic, and inorganic contaminants. The adverse effects of MPs can be based on various factors, such as the chemical composition, dosage, size, color, etc. Although the harmful effects of MP ingestion have been widely reported by the scientific community, further knowledge is still required on MP toxicity [5], and this can be attributed to the lack of standardization methodologies for MP analytical procedures [6].

The presence of MPs and their sampling, extraction methods, and characterization techniques have been extensively reported from the regional to the global scale, especially in the aquatic environment, but in essence, there remains a lack of any harmonization regarding protocols to either sample, extract, or characterize the MPs present in any given environmental matrix. The following constraints arise while working with MPs:

1. The varying size range that has been used by different researchers: The ambiguity in the size range has led to the use of different methods to study the MPs, especially when exposing organisms to MPs in laboratory studies.
2. Another barrier is the unavailability of every technique or instrumentation in all the laboratories studying MPs. Since the scientific horizon is widening at a great pace, new techniques are developed which make standardization an almost impossible task. Furthermore, some research establishments (academic institutions with limited funding, small or newly established research laboratories, non-profit organizations, or research facilities in developing countries) may face financial and resource constraints, making the adoption of efficient and specialized separation approaches prohibitively expensive, leading to the employment of simpler and less precise procedures.
3. The third hurdle is complex environmental matrixes, e.g., working with sewage sludge or wastewater requires many complex steps just to extract the MPs, which, in turn, require additional reagents and chemicals, thus increasing the cost.

Thus, owing to the abovementioned factors and the conditions favorable for a given researcher or group, an array of methods is available, and an absolute standardized methodological approach has yet to be developed and agreed.

The basic frequent approach in any MP study involves sampling, extraction, characterization, and quantification. Despite the quantity of articles published since the UNEP declared MPs to be a worldwide pollutant in 2011, there still remain many knowledge gaps due to the absence of harmonized methodologies. Although MPs were primarily designated as an environmental problem in 2004, it was only in 2020 that the ISO provided a definition for MPs, providing much needed guidance and direction for the research community.

As a result, the science of MPs can be considered to still be in its infancy, now with one defined variable, i.e., the size. However, the main challenge comes with the extraction of MPs, as the plastic behavior changes with the changing environmental profile, and each matrix requires its own set of protocols for a more effective and prudent analysis. This review aims to summarize the various extraction methods that are frequently used in the scientific literature and their associated modifications for a range of environmental matrices: water (fresh water, drinking water, and wastewater), soil, sediment, sludge, biological tissue, and air.

The identification and characterization of the MPs is based on extraction and purification, followed by identification and quantification, including the incorporation of quality-control measures at each step. The extraction of MPs from any environmental matrix is the most crucial step, as it is influenced by the size, shape, and density of both the matrix type and MPs present. It thus follows that a possible differentiation of the extraction processes can be based on the type of matrix being analyzed. The MPs must be isolated from the environmental matrix for characterization and identification purposes. There are many articles highlighting the sampling and the extraction process for complex matrices. In addition, owing to various factors, such as funding, demographics, laboratory resources, and the type of matrix, new protocols are continually developing. Several papers have been published in recent years on the presence, content, distribution, and contamination of MPs in various environmental matrices but not in a systematic manner. To the authors’ knowledge, there are no evaluations that comprehensively and simultaneously illustrate the methodologies for extracting MPs from a wide range of environmental matrices, as most reviews are matrix-specific [7,8,9,10,11,12]. This study aims to classify and incorporate the most recent developments and newest technologies for the different matrices frequently analyzed for MPs in a compact and concise manner. The review is divided into two sections: the first section traces the origin of the original method, prior to modification over time, and the second section provides a detailed account of the different extraction methodologies that are currently in use for different matrix types.

## 2. Methodology

### Literature Search

A wide search of peer-reviewed articles was performed using two online publication databases: SCOPUS and Web of Science. The search was restricted to articles in the English language. For a more systematic review, a bibliometric analysis was performed in this work, using the Biblioshiny platform. Here, more than 80 papers published between 2004 to 2022 are reviewed, with special attention to recent publications. The following keywords, in combination with *extraction* and *microplastic*, were used during the database search: “sediment*”, “soil*”, “water*”, “sand*”, “cosmetic*”, “biota”, and “air”.

For the initial screening, Bradford’s law was used as a criterion to refine the dataset, using the R project and Biblioshiny [13]. Subsequently, the full texts of the articles, selected based on the title and abstract, were examined to shortlist those which met the selection criteria for inclusion in the review. The reference indices of peer-reviewed publications were likewise investigated, and potentially relevant studies not found in online databases were added manually.

This critical analytical method is commonly used to identify the most significant authors, their partnerships, keywords, or geographical locations associated with the research topic.

## 3. Environmental–Analytical Holistic Perspective

The understanding of the fate and effects of MPs can be accomplished via two paths: firstly, through the environmental chemistry approach, in which the effects of various natural matrices (air, water, soil, and biota) are studied; and, secondly, with analytical chemistry, where analytical methods are developed for addressing the challenges related to MPs [14]. The development of new sampling strategies through field-based research, as well as the development and advances in analytical methodologies provided by the development of flow analysis concepts and process analysis strategies, have provided a link between modern instrumentation and social or technological environmental issues in recent years. Regardless of the optimal analytical approach for MP detection, a demanding issue that has been extensively explored is the extraction and isolation of plastic particles originating from varied and complicated environmental matrices. Instrumental technologies and analytical methods have advanced to the point that they now offer a wide variety of tools for determining a broad range of analytes at very low concentration levels.

**Figure 1:**
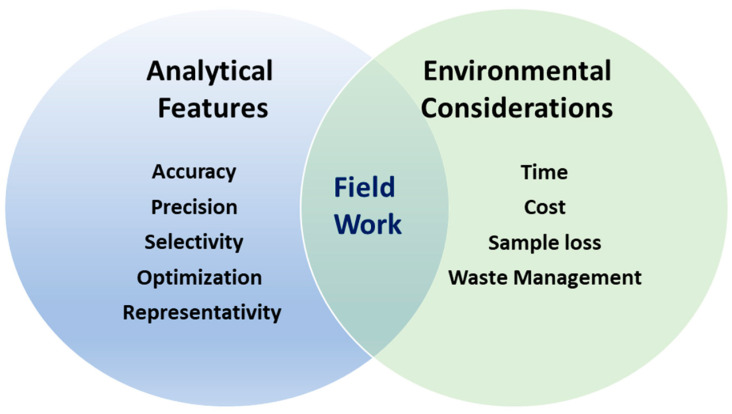
Basic considerations towards building an efficient environmentally friendly analytical approach for MPs.

From an analytical perspective, the defining attributes of a technique that are considered most important to the analyst when applying the most appropriate method in order to obtain meaningful information are accuracy, precision, specificity, and sensitivity [15]. In this context, quantification is critical for sample assessment and comparisons between analytical approaches and methodologies. However, in real time, there are certain practical factors to consider, such as the time necessary to conduct a sample analysis, costs, and user friendliness of the technique, that are essential environmental and analytical considerations. The environmental and ecological perspective is relatively new in analytical chemistry, and in terms of MP research, this should be carefully embraced to ensure the long-term evolution of analytical approaches for the field of MPs (Figure 1).

In this review, new methods and specifications for the extraction of MPs from various matrices are presented in an integrated environmental style, as a problem-solving strategy centered on the development of efficacy for the various techniques, as well as in terms of time consumption and costs. Technological and industrial concerns, as well as environmental, health, and social issues, were identified as challenges for which the scientist must select the most effective instrumental technique and devise an appropriate approach.

## 4. Importance of Matrix Selection

MPs have migrated over the environment and may now be found in almost every compartment on planet Earth, from the highest mountains peak to the deepest ocean floor [16]. The matrix has a considerable influence on the method of choice; therefore, the technique applied can be entirely different for different matrices. An understanding of the composition and properties of the matrix is essential for selecting the optimal analytical approach based on the objectives of the research and available resources in order to ensure harmonization and consistency for standardization purposes. In monitoring studies, the sample matrix for analysis (e.g., water, air, soil, and biota) should be selected depending on the aims of the study, with different matrices providing different information regarding MP contamination in the environment.

It is undeniably difficult to design a novel method for determining which polymers (often degraded) are present and their concentration levels, size, shape, and color in environmental matrices that differ in chemical and physical properties.

In addition, the experimental design must consider the variables of the sampling site that will likely influence the resultant data, including the potential proximity to source, the physical and chemical properties of the matrix, and, in the case of biotic matrices, the feeding niche.

The broad category of environmental compartments includes water, air, soil/sediment, and biota. The main reason for selecting distinct matrices is that the chemical nature of these matrices is heterogeneous, resulting in a wide range of characteristics, functions, and interactions with MPs. For example, sediment samples from beaches, coastlines, benthic habitats, rivers, and lakes are all examples of sediments that can be characterized by their surrounding environment [17]. Most studies on MPs have focused on the aquatic environment [18], especially surface waters, but variations in the hydrodynamic profiles of different water bodies contribute to differences in the abundance and properties of the MP present [19]. For instance, rivers exhibit a unidirectional current with a relatively high average flow velocity, ranging from 0.3 to 1 m s^−1^ [20], while lakes have a modest average current velocity of 0.001 to 0.005 m s^−1^ [21], and groundwater possess a fairly consistent flow pattern in terms of direction and velocity (few meters per day, about 10^−4^ m s^−1^) [22,23,24]. These varied profiles in different environments influence the sediment deposition, presence of flora and fauna, and MP particle movement, thus impacting, in turn, on the sampling and extraction methodology [25].

Many lessons have been learnt from the determination of MPs in water and biota samples, in comparison with soils. Soils are made up of unconsolidated organic and inorganic matter that forms as a result of the interaction of water, air, and organisms on the earth’s surface. It serves as a barrier between distinct environmental compartments in terrestrial ecosystems (lithosphere, hydrosphere, biosphere, and atmosphere). The methods used to extract MPs from soil and sediment may differ dramatically. Organic-matter removal is not often performed when studying mineral-dominated sediments such as sandy beach deposits, but it is usually a requirement for low-energy estuary, lacustrine, or pelagic sediments, and it is also required when evaluating soil. The extraction of MPs from soils is more challenging than from water, given the complex and varying composition of different soil types [26].

The extraction of MPs from environmental matrices has an enormous potential for future method development.

## 5. The Original Methods

Visual sorting, sieving, or filtration are regularly used for separating bigger MPs from fine sediments (mud or silt). Small MPs can be more difficult to separate, especially from finer-grain sediments, depending on the structure (particles, fibers, shape, and size) of the MPs present. The following methods for the separation of MPs from different matrices are reviewed:

1. Density separation: This method was developed by Thompson et al., 2004, and involves MPs from sediment being isolated using a concentrated saline solution [3]. This method is only suitable for polymers with a density lower than the hypersaline brine. Many methods and devices, namely the MPSS, Elutriation techniques, and froth-flotation methods, use the principle of density separation and are discussed later in detail.
2. Oxidative digestion: This method employs oxidizing agents, such as H_2_O_2_, for the removal of natural organic debris, leaving MPs unaffected [27]. This pretreatment is usually followed by density separation or filtration.
3. Alkaline digestion: Various combinations of KOH and NaClO have proven to be effective in the extraction of MPs from biological tissues [28].
4. Acidic digestion: Mixtures of HNO_3_ and HClO_4_ have been primarily used for the extraction of MPs from biological tissue [29,30]. However, concerns have been raised regarding the deleterious effects of these acids on commonly encountered polymers [28].
5. Enzymatic digestion: Among acidic digestion, alkaline digestion, and enzymatic digestion, the latter seems to be the most effective, with no visible impact on MPs during treatment [31].
6. Oil-extraction protocol: Based on the oleophilic properties of polymers, this method was developed in 2017 to extract MPs from environmental matrices, using oil extraction. Mixing sediments with water and canola oil separate MPs in the oil fraction from the sediment settled in the water layer [32].
7. Pressurized fluid extraction: This method was optimized for MP extraction from soils and waste in 2016 [33] and is based on the use of solvents at conditions of subcritical temperature and pressure, principally for the recovery of semi-volatile organics from solid materials. In a first extraction phase, semi-volatile organics are removed using methanol at 100 °C, and MPs are subsequently recovered from the remaining matrix, using DCM at 180 °C.
8. Electrostatics separation: Initially used in 2011 [34], it was only successful for the extraction of spiked sediment samples. However, in 2018, a KWS electrostatic separator was employed to extract MPs from quartz and beach sands [35].
9. Magnetic separation: Introduced in 2019, this method is based on exploiting the hydrophobic surface of plastics to magnetize them to isolate MPs from soil samples [36].

It must be highlighted that the abovementioned techniques cannot be applied to every matrix type and are categorized above based on the sample matrix reported in the literature. A more useful approach for the application of these techniques for different matrix types is presented in the following sections.

## 6. Extraction Methods

Environmental monitoring is essential for ecosystem health, well-being, and protection. Ecosystems are dynamic environments, with characteristic functions, processes, and living organisms, and are subject to the impacts of environmental contaminants, including MPs. In MP research, extraction is the process of separating and purifying MP fragments from the sample medium, while separation involves sorting or dividing plastic into various types, based on shape, size, and color. Water, soil, air, and biota are the four major environmental compartments within which samples can be collected from different ecosystems for monitoring purposes.

### 6.1. Extraction from the Three S’s: Sediment, Sand, and Soil

Due to the vivid, vast accumulations of plastic debris in the oceans and the effects on marine organisms, most attention and research has focused on the marine environment [18]. Whereas plastic waste accumulates in and is transported by water associated with currents and weather conditions, little is known about the role of soils and sediments in the movement, processing, and storage of plastics [37]. Terrestrial ecosystems (sediments, soil, compost, and sand), which are especially prone to plastic contamination, are relatively unexplored and are key drivers in the production and spread of MPs. The extent to which plastics have settled and accumulated in these matrices is critical for understanding their ecological impacts, because the presence of MPs can be detrimental to organisms [37]. Researchers worldwide have used a wide variety of sample processing and analysis procedures, inhibiting comparisons between these different geographical locations and, thus, further impairing the development of a thorough perspective of MPs in soils and sediments.

Sediments and soils are deposited in a variety of sites by wind and runoff and are considered to be long-term sinks of MPs [38]. The best strategy to extract MPs from sediments and soils is a primary concern, and the development of a reliable method for identifying MPs present in these matrices is crucial for better understanding the distribution, mass, and ecological effects of MPs in both marine, freshwater, and terrestrial ecosystems.

The first report on the occurrence of MPs in beach sediments dates backs to 1977, when a survey conducted on 300 beaches in New Zealand observed the presence of virgin polyolefins in the form of virgin plastic pellets [39]; however, it was two decades later when MPs were extracted and quantified for the first time in beach sediments [3]. To date, various methods, ranging from one-step extraction to complex methods based on the oleophilic, electrophilic, or magnetic properties, have been developed. The extraction of MPs from soils is more complex than from sediments and sand, owing to the heterogeneous nature of the soil, and the requirements for the organic-matter digestion methods are more prevalent after the first step of extraction. Soil organic matter is a heterogeneous mix of chemical components, and although the precise chemical composition has remained a source of contention, it is widely recognized that humic compounds are composed of polyphenols, peptides, lipids, and polysaccharides. This review further covers the various methods used and developed by independent researchers and some authoritative bodies.

#### 6.1.1. Pre-Extraction: Sieving and Filtration

##### Sieving

Sieving separates MPs from soil, sand, and sediments based on granulometric fractions and is typically a pretreatment procedure that is used to eliminate particles larger than 5 mm. In several studies, MPs of a certain size were recovered after examination in the field [40], and this technique is recommended when plastic particles are visible to the human eye and are larger than 5 mm. Soils are likely to be moist due to a variety of circumstances, and samples should be dried prior to analysis, which may improve MP recovery when they are bound by organic matter. To ensure clarity and consistency, it is crucial to emphasize the importance of accurately reporting the results, specifying if the work is conducted with wet or dry samples. The reporting of MP quantities should always be a mass/volume of either dry or wet sample, rather than simply reporting the number of MPs by a sample weight.

##### Filtration

Filtration is the technique of separating a solid phase from a liquid phase, using filters of a specific pore size. This method has been employed at many stages of sample processing, such as after density separation or chemical digestion, as well as at the start of the sampling procedure [41]. Traditional filtration procedures, such as filtration under vacuum or membrane filtration, are utilized in the laboratory for analysis. Alumina, ceramics, and polycarbonate are some of the materials used in membrane filters [26,41]. Currently, glass fiber, cellulose acetate, cellulose nitrate, polycarbonate, nylon, and alumina membranes are the most common filter membranes used in MP analysis [42]. All of these filters have benefits and drawbacks; however, there remains little agreement regarding the most appropriate filter material, and, similarly, the filter pore size used ranges from hundreds of micrometers to tens of micrometers.

#### 6.1.2. Density Separation Using Hypersaline Solutions

Density separation is particularly effective for large (kg) samples of sand or sediments [43]. The first step for the determination of MPs in marine sediments is the separation of the sample matrix, which is significantly dependent on the sample type and available resources. The first stage is density separation, in which MPs are isolated from their matrix by utilizing density differences between the extraction solution and the MP polymers. The second stage involves the oxidation of organic matter present, which floats to the surface with separated MPs, frequently making MP extraction and detection difficult. This method of extracting MPs based on differences in their density of the polymer and the flotation solution can be regarded as the primary method for most of the research conducted over the last two decades. It involves placing materials of varying densities in a liquid of intermediate density, where the less dense material floats and separates from the denser material. The average density of sediments and soils varies from 1.70 g/cm^3^ to 2.65 g/cm^3^ [44], and the density difference between the lighter MPs and the heavier sand or sediments can be utilized to separate them. NaCl, being cost-effective and environmentally friendly, has been most widely used. The MSFD Technical Subgroup on Marine Litter has also recommended this method for extracting MPs from intertidal sediments [45]. NaCl, on the other hand, produces a solution with a density of only 1.2 g/cm^3^, preventing the separation of denser polymers. However, because soil particles may greatly adsorb or embed MPs, such a system may require precise tweaking. In the case of soils, for the isolation of MPs, density separation is also the first step, and the digestion of organic matter is also the second step. When the density of MPs is uncertain, higher-density salt solutions are required for MP extraction and flotation, and researchers have experimented with different salts (Table 1).

Density separation methods can be considered an easy approach [8], but the extraction is influenced by the number of factors listed below:

1. Organic matter: More complex matrices, such as soil and sludge, are heterogenous solid combinations of minerals of varied particle sizes and organic matter in various states of degradation. Given the small size and wide variety in shape of the matrix particles, the accurate and absolute extraction of MPs from soil has proven to be difficult. Density separation is a typical method for extracting MPs from soil, but pretreatment in the form of organic-matter removal through digestion methods is required (described later).
2. Wetting agents: Regardless of having the same RIC, studies have shown that the same polymers can include different additives or wetting agents, which affect their density and subsequent separation by considerably reducing the floatability [55]. There are significant differences in the floatability of virgin polymer resins and post-consumer plastic waste [56]. The floatability of the plastics decreases with the increasing concentration of the wetting agent.
3. Hazardous/toxic salts: Certain salts have raised concerns associated with costs and potential hazards, even though they allow for the separation of denser polymers (Table 1). Some regulatory bodies, such as the GHS by the United Nations, discern between two distinct signal phrases that pertain to two levels of the severity of hazard: signal word “danger” is often reserved for the more serious hazardous categories, while “warning” is reserved for the less serious (e.g., CaCl_2_ and Na_2_WO_4_∙2H_2_O). The signal words “danger” is appended to ZnBr_2_, ZnCl_2_, and NaI, chemicals that can cause skin, ocular, and respiratory irritation. The use of NaI has been suggested in various studies because of its reusability, high density, and possible use in combination with separation columns. Based on the number of MPs present in a sample, NaI provides good recoveries, but this is highly dependent on the type of plastic. The use of all of these different chemicals for density extraction procedures has caused uncertainty in selecting the optimal approach.
4. The 3R’s—Repeatability, Reproducibility, and Representativeness: Numerous authors have attempted to establish new protocols for density separation, as the application of those previously described have proven to be inefficient for their particular sample matrix. Furthermore, the efficacy of the various procedures utilized has seldom been compared, and, hence, the data on MP abundance in different matrices may be difficult to compare. The scientific endeavor to develop analytical methodologies for MP extraction has increased in recent years; however, there are frequent contradictions in the literature [57,58].
5. MP dimensions: A recent study tested three protocols for the extraction of MPs from sediments based on density separation (NaCl followed by NaI, NaCl followed by NaI and a centrifugation step, and 10% KOH (m/v)) for three size categories of MPs and observed that the % recovery differed depending on the particle dimensions [59]. Regardless of the methodology or polymer used, MP retrieval was inversely linked to size class, with less particles recovered from sediments for the smallest MP size category [59]. In contrast, 100% recovery was achieved for larger particles, i.e., for those in the range of 2–5 mm, clearly indicating the impact of MP size category on the extraction and the importance of considering these factors when evaluating the data.
6. Overlapping densities: Following density separation using salts with higher densities, some fractions of thermoset plastic types, such as PET/PVC, LDPE/PP, and HDPE/PP, require further separation; e.g., PVC and PET cannot be separated in this manner because their density ranges overlap. The densities of many polymers are similarly close, as in the case of PE/PP, rendering differentiation based on density difficult.
7. Equipment building: Liu et al. created a system that is particularly intended for the extraction of MPs from soil samples, consisting of an acrylic glass cylinder with an aeration disc at the bottom and two rows of 5 mm holes at the top which works on a density separation, vacuum filtration, and a solution recovery step [52]. For ten different types of MPs (PA, PC, PP, ABS, PE, PS, PMMA, POM, PET, and PVC), recovery rates of more than 90% have been observed. However, the separating cylinder is constructed from Plexiglas (PMMA), which is a significant disadvantage, as abrasion generated by stirring coarse soils may result in an overestimation of PMMA contamination in the samples; hence, a non-plastic material should be employed, or PMMA should be omitted from the analysis.

It is clear that all of the density separation protocols referred to in the literature have potential drawbacks, and in order to select the most appropriate technique, the efficacy, the environmental effects, and the cost effectiveness must be considered. High-density salt solutions can be more successful for separating small MP fibers of greater density; however, they are inefficient for MP particles or fragments. This is attributed to the greater surface area of MP particles and fragments and their ability to float in low-density solutions, in comparison with fibers which possess greater density and require a high-density solution for separation [26].

A large proportion of plastic waste comes from PP, PS, and PE classes and accounts for more than 50% of the plastics used in Europe [60]. After evaluating the separation efficiency of several common solutions, Scheurer and Bigalke recommended NaCl as the most appropriate density solution, as polymers with higher densities (PVC and PET) are significantly less abundant in the environment [61].

As a result, regional plastic production and demand must be considered when selecting a separation solution. However, when the overall understanding of the potential MPs present is uncertain, a denser separation solution may be preferable. The use of NaI has been recommended by various studies due to its reusability, high density, and use in combination with separation columns. Based on the number of MP particles present, NaI solution provides good recoveries, but this is highly dependent on the type of plastic present. Several chemical combinations for density extraction procedures have caused difficulties in deciding on the best approach. Recent studies have proposed the use of CaCl_2_ (1.30–1.35 g mL^−1^) as a safer, more cost-effective alternative. However, the large amounts of organic matter that may co-occur with MPs in some environmental samples create an additional impediment to cleanup and enumeration using density-based techniques [32]. However, CaCl_2_ is not suitable for the separation of organic-rich materials [61], as the organic matter flocculates because Ca^2+^ may bridge the negative charge of organic molecules. As a result, the filter may be covered in a thick brownish substance that inhibits the process. Low-density polymers such as PE, PP, and PS can be separated from soil matrices by using deionized water and a saturated NaCl solution, which are inexpensive, readily available, and environmentally friendly.

##### Recycling of Salt Solutions

Recycling, reuse, and reclamation of hazardous waste is required for both expensive and environmentally toxic density separation solutions. Some methods of recycling salts for density separation have already been documented [62]. The reuse of NaI has been extensively researched, and it can be recycled up to ten times through rinsing and evaporation stages, without any chemical contamination and significant loss, in a cost-effective manner (3.7 EUR/kg). Therefore, the use of NaI is recommended, as it is environmentally friendly and can be recycled multiple times, provided that it is not used with a cellulose filter. The recycling process described allows for the recovery of 95% of the NaI salt after each usage [63]. In a recent study, NaBr was recycled five times during the extraction of MPs from soil, with a recovery of more than 90% [52]. ZnCl_2_ may be reused at least five times, while maintaining an efficiency of more than 95%, and this salt solution is considered the most cost-effective approach for isolating MPs from aquatic samples [62]. A saturated salt solution of K(HCOO) is reusable and may be filtered after use for the density separation of water samples [64]. In the Air-Induced Flow technique, the use of NaCl for pre-extraction to reduce the initial sediment sample bulk and the subsequent use of NaI for flotation of MPs were shown to be efficient for extracting common polymer types from marine sediments, including higher-density polymers [65]. The NaCl was used for six repetitions of the pre-extraction step, and NaI was reused up to five times [65]. Whilst most studies considered cost and material savings, it is vital to note that recycling and storage involve additional labor, materials, space, and energy costs.

##### Advancements in Density Separations: New Methods and Customized Sediment Separators

The innovation of density separation instruments is also improving the capabilities of researchers to separate low-density MPs and MPs < 500 μm [66]. To overcome the limits of the separation solution and improve separation efficiency, multistage flotation-based separation techniques have been adopted which are based on the relative low density of plastic. An effective approach for removing MPs from soils using the flotation principle necessitates the consideration and balancing of environmental impacts and operational expenses, both of which are dependent on the stability of the flotation solution [66].

The MPSS system employs pine oil in conjunction with a froth conditioner to increase wetting, lower surface tension, and facilitate the detachment of plastics from sediment in deionized water, resulting in low recovery rates (55%), particularly for high-density plastics [46]. This apparatus can separate large-volume samples and analyze up to 6 kg of sample at the time. MPs have been successfully isolated from estuarine silt and clay, as well as coarse beach sand, using this procedure.

The sediment–MP isolation unit with a 95.8% extraction effectiveness for varied density MPs uses a plexiglass cylinder with a jagged edge overflow structure as the air flotation unit [49], Han et al. constructed a device for recovering MPs from soil and sediment samples, with a typical recovery rate of 90% or greater [67]. The JAMSTEC MP sediment separator is a compact portable glass separator which uses NaI as the floatation media and is simple to clean and reuse, facilitating the rapid separation of MPs from sediments, with recovery rates ranging from 94 to 98% [68].

The standard decanting method, for example, the use of a beaker, is basic in design, but MP adherence to the interior of the container is an issue when the medium is moved, resulting in a relatively poor recovery rate [46]. However, most of the devices mentioned above are complicated and require particular customization, resulting in significant processing costs. Furthermore, for total separation, certain devices have many operating phases, which are labor-intensive and time-consuming, as only small quantities of sample can be treated at one time. Furthermore, using large amounts of flotation liquid comes at a considerable expense and poses an environmental risk.

#### 6.1.3. Elutriation

Elutriation is a method that employs a stream of gas or liquid to separate particles depending on their size, shape, and density in a direction that is usually opposed to the direction of sedimentation. The basic concept came from the realm of biology for extracting meiofauna from sediments. However, the extraction of MPs is more challenging than that of live organisms, requiring the calculation of fluid velocity as a function of particle and fluid characteristics. Sample preparation, on the other hand, may be time-consuming and requires pre-separation into the necessary size ranges. Claessens et al. modified this approach to extract MPs as a first step from beach sands prior to density separation, using NaI [69]. A continuous flow of filtered water through a 15 cm column containing a pre-washed sediment sample (500 mL) agitates the sample from below, dislodging the lighter particles trapped between sediment grains. The MPs are trapped in the overflow, using a small filter at the surface. Although NaI is an expensive salt, by incorporating this elutriation phase, the amount of NaI required is reduced by at least 97%.

In a similar study, Zhu et al. designed an elutriation system based on the Claessens’s device to maximize MP recovery by varying the water flow and column diameter. Reducing the column height to 50 cm (from 147 cm in the Claessens’s device) and having a width between 5.06 and 10.16 cm, the device was tested using a control sample of 500 mL of sand and 50 × 5 mm plastic fragments [70]. The system was simple to use and could be assembled using common household items, thus giving it an advantage over other filtering techniques for removing plastic pollution, such as triboelectrostatic separation, which does not operate in humid environments such as those found near beaches.

Though the authors optimized the flow rate of water to ensure maximum extraction effectiveness, the sand-recovery yield was still high. To address this short-coming, a pre-size fractionation (63 μm–2 mm) of the sample could increase the method’s adaptability. Based on this, Kedzierski et al. used a granulometric approach (particle size subdivision) to optimize the elutriation process, with advantages including (1) the capacity to handle a large quantity of sample in a single run, (2) excellent recovery and viability, (3) speed of operation, and (4) repeatability. A numerical model to determine the elutriation velocity of the fluid was developed, and it was found that in order to limit sand suspension during elutriation, the particle velocity (based on duration of elutriation and height of column) must be tailored according to particle size [71] (see Table 2). This approach, however, was invented and refined solely for sandy sediments, and its efficiency is anticipated to be poorer in fine and/or organic-matter-rich sediments that can agglomerate and/or react with plastics [72].

#### 6.1.4. Pressurized Fluid Extraction

PFE has been presented as a viable solution in the quest for alternatives to extract MPs from soils, sediments, and wastes [33]. The particle size of the MPs has little bearing on this separation approach, and even submicron particles can theoretically be studied [33]. A solvent extraction approach has been reported for detecting MPs and was first introduced in 1995 by Dionex Corporation. It was recognized as an official USEPA method for detecting persistent organic contaminants in solid samples and has been routinely employed for complex matrices. This technique uses organic solvents to extract solid or semi-solid materials with high pressures (3.5–20 MPa) and elevated temperatures (313–473 K), thus retaining the organic solvents in a liquid form above their boiling point while increasing the kinetics of the extraction process once the temperature has reached the certain threshold [73]. This solvent extraction process enables matrix removal and MP enrichment to be completed in a single fully automated stage; however, only a few studies have employed this technique for the extraction of MPs.

MPs from industrial soil and municipal waste material have been extracted using PFE in combination with gravimetric quantification [33]. This procedure involves two steps: static extraction, followed by dynamic extraction, and to eliminate all semi-volatile organic substances (fats and oils), methanol was used at 100 °C during the first extraction phase. The MP fraction was recovered using DCM at 180 °C in the second extraction from the residual matrix. DCM extracts were collected, evaporated to dryness, and gravimetrically quantified. This approach performed well for several plastic types, including PE, PVC, and PP, with average recoveries ranging from 84% to 94% despite the presence of a wide variety of physicochemical interferences [33]. Although plastic particles as small as 30 µm were successfully retrieved, this approach had the following drawbacks: (1) it is limited in its ability to offer size information; (2) the method is destructive, leading to morphological changes in the MPs and, thus, hampering the studies to identify source contribution; (3) failure to provide information on MPs > 30 µm; (4) residues from materials containing various types of MPs will contribute to the acquirement of suitable FTIR spectra, which may necessitate spectral deconvolution skills or sophisticated methods to identify constituent polymers; and (5) the use of a highly toxic chlorinated solvent (DCM) with a very low boiling point. In addition, DCM is a strong polar solvent that can dissolve, at least partially, most plastics but also a large number of natural organic compounds. PFE extraction of sediment using DCM results in a black-brown solution, requiring a multistep purification procedure, decreasing the chances of recovering and identifying MPs of different types.

PFE coupled with GC-MS, using the less toxic and less volatile THF, is an alternative for the quantification of the most abundant MPs (PE, PP, and PS), reaching efficiency levels of 80% when applied to soil and sediment samples [74]. Pyr-GC-MS is a destructive technique based on polymer pyrolysis, which breaks chemical bonds and forms low-molecular-weight moieties from the non-volatile polymer. Thermal degradation products can be cryo-trapped, sorted, and identified based on their mass spectra. Identification is accomplished by comparing the retention time and mass spectrum to polymer standards or by using spectral libraries. Even though this technique has the benefit of high selectivity and specificity and provides a simplified overview of polymer types represented by a common chemical backbone exhibited by basic polymer clusters when a detection threshold is exceeded, it requires a high level of equipment maintenance because the relatively heavy moieties resulting from polymer breakdown may condense in the capillary between the pyrolysis chamber and the GC, causing blockages and cross-contamination [75].

More recently, this technique was further optimized for the analysis of MPs in biosolids by using double-shot Py-GC–MS, and the method required no pre-extraction cleanup phase or sample pretreatment. PE, PVC, PP, PS, and PMMA mass concentrations ranged from 0.1 to 4.1 mg/g dry weight (dw) across all samples, with a total plastic concentration of 2.8 to 6.6 mg/g dw [76].

#### 6.1.5. Magnetic Separation

The magnetic field, which is a controlled force source, has received a lot of interest in separation research, and when looking for suitable removal methods, one of the most easily scaled ways is the use of magnetic adsorbents. This employs magnetic seeds and acid in conjunction with an external magnetic field to increase separation speed. Polymers are hydrophobic; thus, hydrophobic magnetic materials can be utilized to adsorb and subsequently collect them for removal.

Synthesized hydrophobic iron nanoparticles (Fe-NPs) that bond to plastic through silanization and facilitate magnetic recovery have been described as a new approach for the magnetic extraction of MPs from sediment and water samples [36,77]. This technology is based on the usage of tailored Fe-NPs that bind to polymers and allow magnetic recovery, and these customized NPs, which possess a high surface-area-to-volume ratio, are treated with a silane with extended hydrocarbon tails to improve hydrophobicity and sorption to the MPs. Magnetic extraction was shown to be more effective in removing small MPs, and the recovery rate for sediments was poor, because soil particles prevent Fe-NPs from colliding with MPs due to the relatively lower surface-area-to-volume ratio of the MPs. Furthermore, if lipophilic chemicals or biota are present in soil samples, the nonspecific binding of nanomaterials will greatly limit the impact, and, as a result, this technology may be more suited to post-density-separation or digestion stages and for drinking-water treatment. In this regard, relatively high recoveries were obtained (93% for water samples, and 78% for sediment samples), and the technology may be applied to future MP research.

Small MP particles may be recovered successfully from dilute solutions by using magnetic filtering [78], and this procedure does not modify the sample’s structural integrity; however, many knowledge gaps remain, and as a result, most studies have emphasized the use of this technique as a pretreatment rather than as a solitary separation approach.

#### 6.1.6. Electrostatic Separation

Electrostatic separation is a potential approach that has been used in the processing of plastic waste. A critical aspect in attempts to simplify MP separation is that the sample bulk be lowered, and the biological components must be eliminated without affecting the particle characteristics. A reduction in sample mass would result in a large reduction in the amount of chemicals required for subsequent processing. Based on this theory, Felsing et al. proposed the Korona–Walzen–Scheider (KWS), a device used in the recycling industry that takes advantage of the electrostatic characteristics of plastics [35].

The idea behind this method is based on the varied electrical conductivity of the sample particles, with mineral particulates in soil and sand being more conductive than polymers in general. A high-voltage electrical field (max 35 kV, DC) between the grounded drum and an above-mounted rake-shaped electrode charges the particles.

Mineral particles that are more conductive discharge faster and leap off the drum, with the separating flap directing them into the “sediment container”. Less conductive polymers discharge more slowly and remain stuck to the revolving drum, only to detach later and fall into a separate collection container. Here, the use of well-dried and unconsolidated samples is essential; otherwise, the separator will not be able to separate the components because the presence of water alters the electrostatic behavior of particles, and this is also a time-consuming step lasting up to few days.

Electrostatic separation developed as an environmentally benign method among all the separation procedures since it permits the generation of fractions enriched in the elements of interest from biomass particles ranging in size from 10 to 500 µm, but in a recent validation study, some of the drawbacks of KWS were highlighted. The sample-drying process is tedious, and parameters such as the humidity prior to freeze drying is difficult to manage. Another limitation is in the soil-observation data indicates that the presence of tiny particles and agglomerates (as is characteristic for soils) restricts the use of KWS. The electrostatic separation of MPs needs further treatment processes, such as density separation and digestion. However, one crucial element to consider when comparing electrostatic separation experiments is that the mineral composition has a significant influence on the recovery rates [79]. Electrostatic separation does not require any chemical treatment, although it is best suited for large samples; however, sediment loss is a drawback.

#### 6.1.7. Oil-Extraction Protocol

The oil-separation method is based on the oleophilic properties of plastic polymers. Oils have hydrophobic characteristics that can assist with the removal of plastics from environmental samples and increase recovery rates. MPs can be retrieved through the water–soil interface since the bulk of soil or sediment particles are hydrophilic. Although recovery rates have varied between investigations, this process has been shown to decrease surface tension and aid in the removal of plastics from sediment samples [41]. Even with high-density polymers, the oil–polymer clusters have a lower total density than water. The non-polar lipophilic component of these long-chain aliphatic hydrocarbon-dominated fatty acids can attach to the non-polar lipophilic carbohydrate surfaces of synthetic polymer fragments in a quasi-micellar way due to their large molecular weight.

Combining the non-toxic nature of oil and the fact it is not dependent on a specific density as most density-based separation methods are, a new method for the separation of MPs from soil that uses the oleophilic properties of polymers was proposed [32]. The method consists of shaking dry sediment with water, adding a few milliliters of oil, and then placing the mix in a shaker for about 30 s to allow the sediment and plastic to stick to the oil. Water is then decanted prior to a vacuum filtration step. Subsequently, the oil layer is filtered, and the filters are treated with reagent alcohol to eliminate any oil residues that may interfere with further examinations. For samples rich in biomass, it has been suggested to perform digestion before the oil-extraction protocol. According to Crichton et al., recovery rates range from 90 to 100% for all seven investigated virgin polymer types, outperforming two density separation techniques with NaI and CaCl_2_. The technique is straightforward, safe, inexpensive, and time-efficient [32]. The method has been recently validated by Crew et al. for studying the variation in the distribution, abundance, and diversity of MPs across the St. Lawrence River [80]. However, this procedure may limit the recovery rates due to one of the following reasons: (1) all the MPs are not transferred to the oil phase and may remain in the sediment; (2) the MPs remain intact on filters and the glass surface while transferring, leading to underestimation; (3) recovery rates decrease while working with real environmental samples; (4) due to the use of reagent alcohol, MPs (particularly PE and PS) have high static electricity during examinations; (5) after the rinsing step with alcohol, some of the MPs remain in the oil, adding to further underestimation [81]; and (6) shaking the sediment with water and oil often results in the formation of an emulsion when samples contain natural surfactants. Such emulsions are often hard to break down and lead to poor extraction yields.

Further research attempted to increase the overall performance of this system by adjusting many phases.

To minimize the shortcomings of the first proposed method, different studies have tested different oils, as listed in Table 3. Without the requirement for oxidation, the oil-separation method can be utilized to extract a variety of soil types. The recovery rate from low-density polymers to high-density polymers is likewise significant, with an average of over 90%. In another experiment, the recovery rate was boosted by adding a drop of olive oil to the NaCl solution, increasing the rates from 64% to 80%. When compared to other salt solutions, oil extraction is both cost-effective, environmentally benign, and broadly applicable to numerous different MP types [82]. It should be emphasized that oil extraction necessitates a detergent cleaning phase [83].

For a wide spectrum of polymers, the optimum oil should have a strong affinity between oil and each micro-polymer. The viscosity, density, and surface tension of the oil determine the oil’s attraction for MPs. The higher the viscosity, the better the interaction between the oil and the micro-polymer. Among the oils listed in Table 3, MPs have been extracted from soil and compost samples, using olive oil [84]. Castor oil has the highest viscosity, i.e., 580 cP, in comparison with the natural plant oils listed. However, when utilizing castor oil, the recovery rate of PS MPs is found to be quite poor, with a value of only 24% of that when using olive oil [85]. The effectiveness of utilizing oil to extract MPs from a solid matrix is heavily reliant on the use of an appropriate agitation rate [85]. A recent study created a highly selective MP separation technique that consists of two phases, both of which include hydrophobic interactions, namely interactions between MPs and oil in the first step and interactions between oil and a hydrophobic/magnetic PDMS-coated nickel foam adsorbent in the second step. This study tested two vegetable oils (sunflower and canola oil) and a mineral oil, highlighting the superiority of mineral oil in extracting MPs from soil [85].

A comparison of the different extraction methods that combine oil-extraction protocol and density separation showed that there is no robust protocol for extracting all types and forms of MPs from fine sediments, and that further efforts to develop a reliable method must consider the interaction of MPs with other particles, as well as the electrostatic properties of MPs [46]. The relatively small sample size of sediment that may be analyzed hampers representativeness, and it is a limiting issue in the application of this technology for sediment samples. Furthermore, the extrapolation of determined MP concentrations to a larger volume increases the uncertainty. Interestingly, oil separation is a time-saving and environmentally beneficial process that warrants further developments as an alternate separation method.

### 6.2. Water Samples

The early evaluations of plastic in the coastal and marine environment were based on debris floating on the surface or immediately beneath the ocean’s surface. Most investigations into plastic contamination have been undertaken in the marine environment, likely because nearly half of all the plastic manufactured has a lower density than saltwater and is expected to float at sea. Furthermore, the NOAA has conducted the only attempt of standardization for MP sampling methodologies which focused on water and sediment matrices [86].

However, this recommended methodology can only be used to determine plastics with sizes ranging from 0.3 to 5 mm, including PE (0.91–0.97 g mL^−1^), PP (0.94 g mL^−1^), PVC (1.4 g mL^−1^), and PS (1.05 g mL^−1^), thus limiting the range of MPs detected in water samples. This technical memorandum, on the other hand, may be viewed as a first step toward the much-desired standardization of sampling and sample-processing procedures for MPs in water and sediments.

Water as an environmental matrix encompasses different forms, including freshwater (surface water and groundwater, including drinking water), seawater, and wastewater.

The distribution of MPs in the water column is driven by their physical characteristics (density, shape, and size) and environmental factors (biofouling, water current, waves, hydrodynamic profiles, and density). Freshwater and saltwater have different densities of 1.00 g/cm^3^ and 1.03 g/cm^3^, respectively, which can contribute to different distributions of MPs in the water column in either system [87].

Wastewater is another category of water that has been investigated for the presence of MPs [88]. Freshwater samples frequently contain greater quantities of natural particles than surface seawater, confounding sample analysis even further. Large amounts of MPs are detected daily in WWTPs, as the treatment process removes MPs only at the aeration stage, and a significant amount of MPs escape through the filter and are released into the receiving waters.

To identify MPs in these complex (i.e., with numerous types of matrices) and organic-rich environmental matrices, appropriate analytical techniques are required. In many instances, a combination of density separation and organic-matter digestion techniques (addressed later) are used to eliminate both the mineral and biogenic substances from the sample [54].

Despite the fact that the sampling processes in freshwater and marine settings are similar, the quantities of organic and inorganic compounds in the samples differ significantly. The extraction of MPs from water sources should be conducted according to the nature of the water; for example, for drinking water and bottled water, simple filtration or sieving, followed by staining, is sufficient, while for samples containing organic matter, digestion methods are required.

- **Water-Volume-Reduction Method**

The volume of water samples collected fluctuates between studies; only a portion of the sample is of interest and is retained for subsequent processing. Filtration is considered the first step in extracting MPs from large-volume water samples.

Size-based separation procedures, such as sieving and filtering, are widely employed to extract MPs from environmental samples, with the aim of reducing the quantity of samples collected. Although the effects of sieving on polymers, particularly aged and weathered polymers, are rarely discussed, sample transfer across the sieves may lead to MP loss, fragmentation, or cross-sample contamination.

Water samples can be initially sieved using 500-mesh sieves. For complex and organic-rich water samples, centrifugation is a useful alternative to sieving as a water-volume-reduction technique; however, the sample processing is time-consuming and inappropriate for marine and estuarine samples that require large volumes.

Centrifugation has been applied to wastewater biosolids, snow, vegetal-rich samples, and biota studies [89]. Following volume reduction, the appropriate approach for subsequent analysis is determined by the source of the water sample.

#### 6.2.1. Clean Water Samples: From Drinking Water to Seawater

Drinking-water supplies, including bottled and mineral water, come from surface or groundwater sources. Almost all drinking-water supplies obtained from surface waters are filtered, and some are further treated.

Adsorption, coagulation, membrane filtration, oxidation, and microbial degradation are the most prevalent ways to remove MPs from these types of samples and require minimal or no pretreatment (in the case of bottled water), and extraction can be conducted via sieving or vacuum filtration. Density separation can be directly applied to samples that are low in organic-matter content, and if necessary, subsequent organic-matter digestion can be conducted.

A fresh concept for identifying the MP polymer type in the field, utilizing density separation with non-hazardous reagents, was recently demonstrated [90], primarily addressing issues faced by scientists working in remote areas and lacking access to analytical instrumentation. This method involves the use of ethanol, distilled water, and sugar combinations to produce a variety of solutions with varying specific densities and polymers that are then categorized based on their ability to float or sink in the specific solutions. This approach proved successful in distinguishing seven common polymers taken from the water and shoreline of Samos, a Greek island in the Aegean Sea [90]. For example, the 7:11 ratio of EtOH/H_2_O can distinguish between LDPE and HDPE based on their floatability in the given solution. This approach can be generally framed by introducing green, eco-friendly, and sustainability aspects, for example, into MP analysis [91].

#### 6.2.2. Wastewater

Wastewater and sludge are more complex samples to analyze due to the dense matrix of organic components, bacteria, and inorganic particles bonded together by biopolymers, which have a high affinity for most polymer surfaces.

Moreover, most studies to date involve varying sample volumes and the use of different sieves, grids, and other sampling devices [92].

Furthermore, different types of polymers are the focus of different studies, and there are many inconsistencies regarding sampling strategies (individual and composite samples). Although many methods for determining the MP concentrations in water and sludge have been developed, many knowledge gaps remain.

Larger plastic particles are efficiently removed during wastewater treatment, while MPs frequently circumvent the treatment process and accumulate in the receiving waters. In addition, a considerable number of WWTPs are situated along the coast, representing a substantial source of MPs to the marine environment [92].

Special attention is paid to MPs originating from PCCPs, which are recognized as an important source of MPs in wastewater and, consequently, in surface waters. MPs are specially designed for sizes between > 0.1 μm and ≤ 1 mm and are added as cleansing or exfoliating agents to various PCCPs (shower gels, facial and body scrubs, toothpaste, nail polishes, etc.). The most commonly used polymer for this purpose is PE [93,94]. The load to the aquatic environment with MPs from this source originates from their application in everyday human activities, whereby they are washed out in wastewater, arriving at WWTPs, or directly discarded into the aquatic environment [95].

Therefore, researchers are applying different procedures to isolate MPs from cosmetic products in order to investigate the potential harmful effects on the environment. Most of the works on this topic were published in the period from 2015, which represents the period when PCCPs were recognized as a source of MPs in the environment [95,96,97,98,99,100,101,102,103,104,105,106].

A similar method with minor modifications was used in studies in which MPs were isolated from PCCPs. Typically, the isolation procedure consists of four steps: (1) dissolving 0.5–10 g of PCCPs in deionized water (150–1000 mL), using stirring, with or without heating; (2) filtration with different filters (Whatman ™ filter paper, coffee filter, Filter-Lab 1242 filter, cellulose filter paper, nitrocellulose membrane, Macherey-Nagel cellulose filters, stainless-steel filters, etc.), whose pore size ranged from 0.45 to 12 μm; (3) rinsing with deionized water; and (4) drying at room temperature or at a temperature of 50–60 °C for 7–24 h or until constant mass [95,99,101,105].

After isolating MPs from PCCPs, further characterization of the particles in terms of size, shape, color, etc., was performed. Furthermore, the particles were applied for the examination of interactions with other constituents, the estimation of the amount of MPs reaching the wastewater streams, and ecotoxicological studies. It was found that MPs isolated from PCCPs have a good adsorption potential towards other pollutants in water, and ecotoxicological tests have shown that they have a potential negative effect [96,101,102]. Although many companies, in last two years, have abandoned the application of MPs in PCCPs and replaced them with more environmentally friendly options, the pollution from these types of MPs is still present in aquatic ecosystems, and studies of their behavior are still necessary.

##### Conventional Wastewater Treatment Systems

To remove particles, organic materials, and, in some cases, nutrients from wastewater, traditional wastewater treatment uses a combination of physical, chemical, and biological processes and activities. According to their level of processing, going from lowest to highest, the general terminology used to describe different degrees of treatment include preparatory, primary, secondary, tertiary, and/or advanced wastewater treatment.

Even though commonly used processes in WWTPs, such as ultrafiltration and membrane filtration, are not specifically intended to remove MPs from wastewater, excellent removal efficiencies are attained.

According to the different stages of wastewater treatment, usually 35–59% of MPs are removed during preliminary treatment, and 50–98% are eliminated during primary treatment. At this stage, the principal method of removing MPs is the skimming and settling of entrapped MPs during gravity separation. Despite the excellent removal efficiency, the large volumes of water processed result in the discharge of MPs in effluent from WWTPs daily [107].

The MP type has also been hypothesized to play a role in removal efficiency during wastewater treatments, with primary MPs being more successfully removed than secondary MPs since the former is often present as microbeads, whilst the latter is more likely to be textile fibers or other debris [108].

##### New Techniques for Microplastic Separation

- *Ferrofluid-based Separation*

Magnetic extraction has been applied for the removal of MPs from wastewater [36], and the novelty of this approach is that, while dealing with small-sized MPs, the extraction efficiency was higher because more Fe nanoparticles could be bound per unit mass of plastic.

The advancement of this technology is nano-ferrofluids, which are colloidal compositions comprising single-domain magnetic nanoparticles in a liquid carrier [109]. This approach has gained the interest of the industrial and academic scientific communities over the last few decades due to the large surface area of the nanoparticles that can efficiently bind contaminants.

The formulation of ferrofluid involved has been tested by mixing a lubricating oil and FA to modify the iron nanoparticles by two independent authors. Zhao et al. modified FA with iron nanoparticles via a co-precipitation method for the removal of PS nanoplastics from sewage as a new eco-friendly adsorbent. A strong interaction was observed between Fe-modified FA and PS nanoparticles, highlighting the reusability of these new magnetic materials up to four times [110]. However, this method was only tested for PS nanoparticles, and tests for MPs still need to be conducted [110]. Similarly, Hamzah et al. tested four types of oil (cooking oil, used cooking oil, lubricating oil, and used lubricating oil) for the production of ferrofluid to isolate MPs from greywater. These tests were based on the hypothesis that if oil can trap all MPs, magnetite can remove a significant amount of the MPs suspended in the water. The results indicated that the production of ferrofluid using lubricating oil resulted in the maximum MP extraction efficiency (99% PET removed from synthetic WW and 64% PET removed from greywater), and this outcome is related to the stable features of these comprehensive mixture of hydrocarbons [111]. This method requires further validation, as the size of the PET was 2 mm, and, thus, further tests should involve MPs that are smaller in size.

- *Photocatalytic micromotors*

Micromotors are miniature self-propelled machines that have caught the interest of academics all over the world due to their applicability in oil removal, metal/metalloids removal, and organic-matter degradation. Wang et al. demonstrated the robust photocatalytic removal of PS MPs from wastewater by using a gold-decorated TiO_2_-based micromotor under UV light [112]. As they are photocatalytic, these micromotors can move in water due to the photocatalytic reactions on the particles, thus requiring no fuel.

Additional methods have been explored, including the use of bioinspired molecules (made up of a framework of organic and inorganic molecules) for MP removal. This involves an inclusion unit; an alkoxysilyl functionalized bioinspired component; a capture unit to catch MPs and nanoplastics in the inclusion unit, based on hydrophobic and Van Der Waals interactions; and a sand filtration device for the removal of trapped micro- and nanoplastics. As no experimental work was conducted on this technology, this method lacks practicality; however, in the future, exploitation of this adaptable host–guest system to protect filtration membranes against micro- and nanoplastics can be considered [113].

### 6.3. Biota

The bioavailability of MPs to marine species is of significant environmental concern, and the extent to which organisms consume MPs is critical for determining and monitoring the environmental status regarding plastic pollution. Several studies have indicated that MPs are consumed by marine biota [114], and therefore the reliable extraction, identification, and quantification of MPs in biotic tissues is critical. In recent years, a wide variety of procedures, including dissection, depuration (in particular cases) [115], homogenization, and digestion of organic matter, have been proposed and modified, with the selection of one or more approaches being primarily driven by the organisms under investigation.

Digestion is the most commonly used method for MP isolation from tissues, and many digestion methods have been devised, involving the use of alkaline reagents such as NaOH or KOH, acids [116], HNO_3_, HCl, and HClO_4_; enzymes [117] and oxidants [118] such as peracids; and H₂SO_4_ (Table 4).

Several methods for dissolving organic material with acids have been introduced into MP research [28,119]. Strong oxidizing agents have usually been used in digestion; however, synthetic polymers can be destroyed or degraded by these chemical treatments, especially at higher temperatures [120]. Acids can destroy biogenic substances with a high level of efficiency (94–98%), but they can also dissolve polymers [28]. Although acidic digestion affects numerous polymers, it should be avoided and utilized with extreme caution when other approaches fail.

KOH is effective at breaking down fish tissue, but saponification frequently complicates traditional KOH digestion methods in lipid-rich tissues [121]. This reaction results in the formation of a glycerol and fatty acid (soap) solution, which can encase MPs, preventing their recovery, and clogging the filter. To reduce the impact of lipids on MP extraction, the introduction of additional steps in the procedure (e.g., ethanol) that do not interfere with the MPs is necessary.

While enzymatic digestion is a new separation approach in MP research, chemical treatments are the most used methods due to their lower cost and relatively good recovery rates (explained in detail in the next section).

**Table 4:** Digestion methods for extracting MPs from marine biota.

S. No.	Reagent	Organism	Exposure Time	Temp	Ref.
1	Pancreatic enzyme and tris	Bivalve Tissue	12 h	37.5 °C	[117]
2	HNO_3_:H_2_O_2_ 65%:30% 4:1 by volume	Mussel	30 min	50 °C	[116]
3	10 M NaOH 5:1 (*w/v*)	Mullet	48 h	60 °C	[122]
4	HNO_3_: NaClO 1:10 by volume	Herring	5 min	Room temp.	[123]
5	H_2_O_2_ (30%)	Bivalves	24–48 h	65°C	[118]

Another popular method for examining MP ingestion in marine biota (larger organisms) is dissection of the gastrointestinal tract and the subsequent chemical degradation of the tissue, leaving only indigestible remains, including putative MPs [124].

### 6.4. Organic-Matter Removal

One of the greatest challenges for MP detection is distinguishing between plastic and naturally occurring particles. Removal of the organic matter from complex matrices is indispensable. This step facilitates the extraction and isolation of the target MPs, and without organic-matter removal, remnants of shells, plankton, vegetation, or other debris still remain, thus increasing the time involved in optical and spectral analysis.

As the size range of MPs varies from anywhere between 1 mm and 1 µm, smaller-sized particles are more pronounced and have greater impacts due to their greater surface-to-volume ratio, and MPs with small diameters are more sensitive to digestion methods than larger ones.

For mineral-dominated sediments such as coarse sandy beach deposits, organic-matter removal is often not necessary, but it is usually required for low-energy estuarine, lacustrine, or pelagic sediments, as well as for soils, sludges, or composts.

Organic-matter removal is usually conducted after density separation, when the sample has been reduced to only MPs and lower-density organic materials; however, in some cases, it is performed prior to density separation [125]. Although the order in which these steps are completed has no effect on MP recovery efficiency in spiked samples, it may result in greater MP recovery in soils where the MPs are entrapped in organically bound soil aggregates. Thus, the nature of the sample, the analyte, the availability of reagents, and the equipment typically play a critical role in the selection of the digesting technique, facilitating selection of the optimal process for the largest yield of extractable MPs.

The following factors must be optimized: (1) digestion reagents (acid, base, oxidants, and enzyme); (2) temperature of digestion (room temperature and high temperature); (3) digestion period (hours and days); (4) digestion steps (one, two, or more); and (5) concentration of reagents.

**Figure 2:**
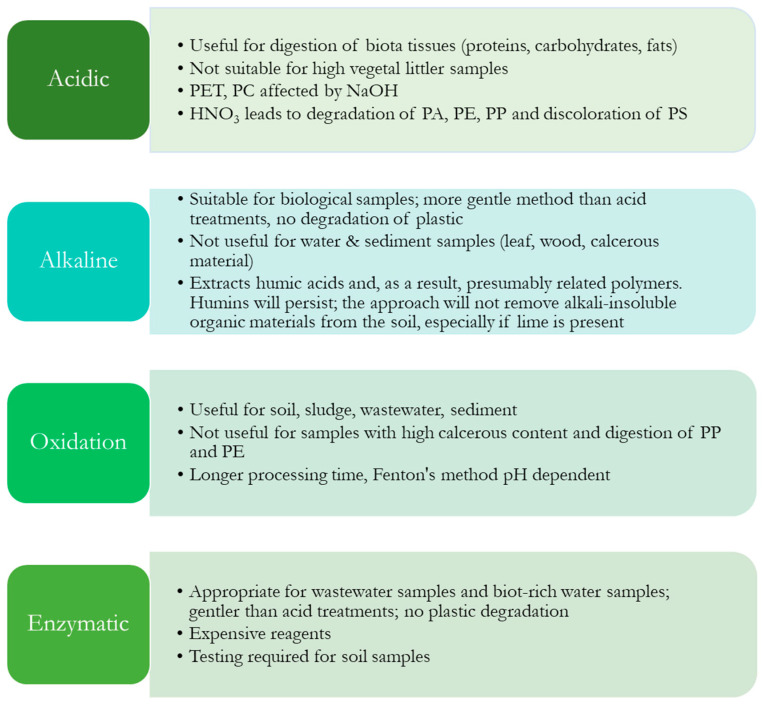
Summary of digestion methods used for removal of organic matter.

Ideally, any methodology employed must not change the MP properties, such as its weight, quantity, or shape (see Figure 2 for a summary of digestion methods used for removal of organic matter and Table 5 for organic-removal methods used in various studies).

#### 6.4.1. Acidic Digestion

To decompose organic materials in various samples, acids such HNO_3_ and HCl have been used in open or closed systems, in combination with a high pressure and temperature, to oxidize organic matter present. The matrix influences the efficiency of biogenic organic matter decomposition.

Concentrated HNO_3_ (high temperature) has been shown to effectively remove organic matter from biotic tissues [65,119,130], mainly comprising carbohydrates, proteins, and fats. However, the use of HNO_3_ was found to be unsuitable when MP extraction was performed on mangrove vegetal litter, as it degraded the PA, PS, and nylon fibers that were present and tended to change the color of certain plastics, such as PE, PP, and PVC [127].

When HNO_3_ is combined with other acids, its oxidizing power can be enhanced; e.g., although HCl is a non-oxidizing acid, when used independently, it becomes a potent oxidizing agent when combined with HNO_3_ (aqua regia) in a certain proportion (HCl:HNO_3_, 1:3). This is because the products of the reaction, nitrosyl chloride and chlorine, are powerful oxidizing agents. Aqua regia has been reported to be less aggressive for MP particles and fibers than HNO_3_ (at similar concentrations). Most regularly used plastic types (PE, PP, PET, PVC, and PS) have been shown to be resistant to a 5% HCl solution, except for PA, which is degraded, even in diluted HCl [130].

Acidic treatment has the advantage of removing some inorganic particles, such as carbonate (particularly in sediments), and as a result, acidic digestion is not recommended for biogenic organic material in sediments and water samples, unless the sample matrix has a high concentration of calcareous compounds [131].

Sulfuric acid has also been used for acidic digestion to avoid precipitation of calcium carbonate in sediment samples in combination with H_2_O_2_ to form peroxymonosulfuric acid, which oxidizes organic residues, but the sample had to be stirred for one week [132].

In spite of the above-described shortcomings of acidic digestion, the ICES considers the use of acids to be the most suitable method for the digestion of tissues, as it not only digests the tissues but also removes all other organic material present, leaving only silica (e.g., sand particles) and plastic particles. The approach suggests using a 4:1 combination of HNO_3_ and HClO_4_ as digesting agents and has marked the importance of further improvements, as nylon fibers are degraded by this method [133]. It is worth noting that there are many potential hazards associated with the use of HClO_4_.

To improve the extraction of MPs, a new thermoanalytical approach has been employed to remove organic matter, followed by an acidification procedure that removed inorganic carbon (e.g., carbonate minerals and shells), using >85% H_3_PO_4_ [134]. Nevertheless, as H_3_PO_4_ is reported to be a poor digestant [135], and combined with the fact that it is an extremely strong oxoacid, the effect on the MP chemical integrity must be further investigated.

#### 6.4.2. Alkaline Digestion

Alkaline digestion is another experimental approach for the removal of organic matter; however, depending on the reagent used and the incubation temperature, the results may vary. For example, Hurley et al. reported substantial degradation of PET and PC MPs after digestion with NaOH (10 M) at 70 °C. This impact was reduced when 1 M NaOH was used, with the development of ‘peeling’ on the surface of PET and the development of a matte texture on the PC [136].

Alkaline hydrolysis is efficient at degrading proteins, and it is widely used to remove MPs from biota [137]. The optimal alkaline approach for extracting MPs from biota has been identified as 10% KOH at 60 °C. Cellulosic and chitinous material, on the other hand, are resistant to KOH and NaOH treatment and may be necessary for sludge and soil samples. Furthermore, alkali-insoluble humins are frequently the most common organic portion in soils, and this explains why NaOH and KOH have poorer removal efficiencies [136]. Another innovative strategy is to eliminate cellulosic-rich sample components by using a urea solution with thiourea and NaOH; however, this method requires the use of MP-degrading NaOH. In addition, this method requires another oxidative digestion step. Moreover, it has been reported recently that a mix of urea:thiourea:NaOH can dissolve cellulosic materials, with both advantages and disadvantages [138].

According to the findings of Pfeiffer and Fischer in 2020, digestion processes that use alkaline protocols are also less suited for use on sediment and water samples [131]. These samples are distinguished by larger quantities of cellulose, hemicellulose, and lignin from plant remains that are only slightly modified by alkaline digestion. Furthermore, NaOH and, to a lesser extent, KOH were shown to partially degrade some MP types, such as PET, which makes up the majority of fibers in environmental samples [139].

A mixture of 30% KOH and NaClO in a ratio of 1:1 has been reported to be most appropriate due to its widespread availability and excellent digestive capabilities, and it is purported to be a rapid, economical, and effective digestion approach for sediment samples [28].

#### 6.4.3. Oxidative Digestion

Oxidizing agents are attractive possibilities for the most efficient digestion of a variety of sample matrices as it can digest grease, cellulose, fat, and chitin shells [140]. The most used oxidizing reagent is H_2_O_2_, which may also aid in the filtering and identification of MPs [141]. However, the overall effectiveness of H_2_O_2_ as an oxidizing agent for the determination of MPs in biota has been questioned.

Cole et al. discovered that only 25% of biogenic material was eliminated after 7 days of treatment with 35% H_2_O_2_ at room temperature [31].

Some studies have used higher temperatures during peroxide oxidation to shorten reaction time; e.g., Sujathan et al. utilized 30% H_2_O_2_ at 70 °C to reduce the reaction time to around 12 h. While 70 °C is lower than the continuous operation temperatures for the majority of popular polymer types, the authors cautioned that PMMA particles may be damaged. A modified strategy employing lower temperatures may address this problem; however, the impact on the reaction time must be evaluated [142].

Temperature regulation plays a significant role in digesting the unwanted matter; e.g., at room temperature, the use of oxidants such as H_2_O_2_ has only a modest influence on synthetic polymer weight and size but can cause discoloration, while an increase in temperature to 70–100 °C induces a considerable reduction in the weight and size of PA fibers [127,131,132,133,136].

The wet peroxide oxidation method is among the most successful procedures for digesting organic matter in soil, sludge, wastewater, and sediments [81]. Furthermore, the reaction is faster than standard H_2_O_2_ oxidation, often requiring less than 1 h for wastewater samples. It entails the use of a combination of H_2_O_2_ and ferrous ion, Fe^2+^, known as the Fenton’s reagent. This method has been recommended by the NOAA and involves 30% H_2_O_2_ and heating the sample to 75 °C.

The ferrous ions catalyze the breakdown of H_2_O_2_ and the formation of radicals, which function as powerful oxidants. Plastics can tolerate peroxide oxidation, whereas organic matter degrades at 75 °C [86]. The protocol’s viability is mostly determined by the management of the pH (optimal at 3.0) and the reaction temperature (40 °C). When the pH range surpasses 5–6, this approach may be deleterious for carbonate soils owing to the development of Fe(OH)_3_ precipitates [143]. However, when using H_2_O_2_ or Fenton’s reagent for the digestion of organic-rich materials, significant temperatures may arise due to exothermic oxidative processes.

Temperatures exciding 40 °C or 50 °C are not recommended [131], and this can be achieved by lowering the concentrations and/or immersing the sample beakers in a cooling water bath. Fenton’s reagent, alongside NaCl, has been reported to reduce organic matter in estuarine samples by 98%, with almost no effect on polymers [89].

The duration of the oxidizing methods is crucial, as it governs how long the matrix is exposed to the oxidizing agent. Exothermic reactions can be accelerated by increasing the period of exposure, which increases the extent of solubilization of the polymer of interest from the biological matrix. The normal time to complete a wet digestion via open vessel digestion is 1 to 2 h; however, this might vary depending on the circumstances. As a result, it is critical to select the most effective digesting temperature and duration to obtain the best MP recovery.

#### 6.4.4. Enzymatic Digestion

Enzymatic degradation is another promising method for removing organic matter which employs physiologically specialized techniques for hydrolyzing proteins and breaking down tissues [144]. Enzymes, as opposed to chemical-digesting procedures, assure no loss of MPs, degradation, or surface alteration and are also less hazardous to human health [145]. They are mostly employed alone, but sometimes they are used in combination with other digestion methods. Proteins, lipids, and carbohydrates are examples of organic materials that can be precisely eliminated [144]. Cole et al. proposed the use of a single enzyme (proteinase K) to separate MPs from saltwater samples containing a high concentration of planktonic species [31]. Although a high level of separation was achieved, a notable disadvantage of the method is the high cost of the enzyme that is often utilized in molecular biology procedures. Meanwhile, low-cost enzymes have been effectively utilized to remove plastic particles from mussel tissue samples, such as lipase, amylase, chitinase, and cellulase, throughout the breakdown process [146].

Taking all the possible digestive methods into consideration, it is vital to note that the composition of the matrix must be given preference, as different reagents behave differently with the constituents of any given matrix.

For example, the procedure applied for the removal of organic matter in marine organisms or biogenic tissues may not be appropriate for samples where vegetal matter predominates, such as in soil or sediment, as they consist of humic substances, which are complex polymers created from the breakdown products of plant and animal leftovers.

The sediment matrix has a more or less similar composition to soil, except it has a higher calcareous concentration and is often found in shallow waters near land; however, even among marine and freshwater settings, each sample is unique.

Calcareous compounds show very little reactivity towards oxidizing agents and bases but are easily dissolved by both HNO_3_ and HCl [131]. In order to achieve the optimal digestion outcome, while also considering the best possible preservation of the targeted plastic particles, all methods are recommended to be tested for extraction efficiencies in laboratories before and during use, as efficiencies can vary between personnel and the method used. This frequently necessitates a multistage approach involving the successive application of various digestive solutions. In most cases, interim washing is required between the individual steps, thus increasing the likelihood of contamination.

According to the literature, any method that uses aggressive reagents at high temperatures and prolonged digestion periods will harm MPs.

These studies that we reviewed may be accurate in terms of polymer identification, but their yield may be skewed in terms of size estimates; thus, different chemical treatments can degrade or damage synthetic polymers in different ways, with enzymatic treatment causing less damage; however, it is not widely employed due to the costs involved. The chemical resistance data [130] for individual polymers can be referred to prior to the selection of chemical reagents. In Table 6, a part of the chemical resistance is shown [130]. For other reagents, the complete chart can be found elsewhere [130]. In Table 7, the costs of reagents used in organic-matter removal are shown.

### 6.5. Air

There are numerous publications on MPs in the aquatic environment, but very few studies address MPs in the atmosphere. Recent publications are dedicated to MPs in the atmosphere, where MPs have been detected in the air of urban, suburban, and even remote areas. These studies suggest that MPs can be transported in the atmosphere over long distances, far from the MPs’ source [147,148]. This leads to the dry or wet deposition of MPs in remote ecosystems.

Synthetic fibers or fragments from clothing and textiles, abrasion from rubber tires, construction materials, and road dust are considered the most important sources of primary MPs in the atmosphere [149]. Due to their small size and low density, MPs can be easily transported and transferred by wind to other environmental compartments. Therefore, atmospheric MPs can be a source of land and aquatic MP pollution. Other sources of MPs in the air can include incinerators, landfills, construction materials, industrial discharges, and particulate emissions from vehicles [150,151].

MP fibers and fragments are the most commonly detected forms in the air, and their size and shape can affect the transport of MPs through the environment. Airborne fibers may originate from textiles, while fragments may originate from the breakdown of larger plastic products. The size of atmospheric MPs identified to date ranges from 2 μm to 1 mm, with PE, PP, PA, PS, and PET being the predominant polymers [152]. Meteorological factors have a great influence on the dispersion and content of MPs in the air. Elevated concentrations of MPs are detected in terrestrial and aquatic ecosystems during winter, suggesting that precipitation and rainfall have an impact on the concentrations and deposition of MPs in air at sampling sites [151].

MPs in the atmosphere originate from anthropogenic activities and can be divided into industrial, domestic, and agricultural sources [153]. Urban road traffic and road dust from tire abrasion are considered an important source of atmospheric MPs in urban areas [154,155]. Weaker air currents in urban areas also contribute to higher atmospheric MP concentrations than in rural areas. More suspended MPs were found indoors than outdoors [156]. Textile fibers from clothing, bedding, upholstery, and curtains are a major source of MP indoors and are released into outdoor air through air exchange [157].

The sampling and analysis of airborne MPs is a complex and multistep process, with the techniques used varying significantly between studies. There are two common sampling methods for collecting airborne MPs: active pumped samplers and passive atmospheric collectors. Suspended aerosols in active sampler are collected using a pump-driven sampler that captures the aerosols through a filter membrane. The air is simultaneously pumped and filtered, and the MP particles are retained on the filters. The active sampling method can quickly collect atmospheric MP particles in outdoor or indoor air, but it requires electrical power. With passive methods, atmospheric precipitation (wet and dry deposition) is collected via a funnel into a glass bottle, and MPs are filtered from the collected atmospheric precipitation. The passive sampling method is easy to use and is suitable for sampling in remote areas without access to electricity or for long-term continuous collection. The different sampling methods for MPs make a direct comparison between atmospheric fallout studies difficult [158,159,160]. The majority of research published to date on atmospheric MPs has used a passive collector (total deposition). Passive samplers provide a site- and time-specific amount of MPs falling on the surface (urban road surface, rural field, or remote mountain). Subsequent processing methods depend on the type of sample and identification methods. Digestion with acids, bases, or oxidizing agents can remove impurities that are free or bound to MPs. CaCl_2_, ZnCl_2_, or NaI solutions are used for density separation. Glass microfiber filters are most commonly used for filtration because they are very effective at removing small particles from the air. In general, water samples obtained by wet or dry deposition must be filtered. For air samplers, the filters can be analyzed directly. The particle size distribution, shape, color, surface morphology, and polymer composition of MPs in the atmosphere are generally measured, and μ-FTIR is commonly used to determine the polymer composition of MPs in air samples [161]. Other methods, such as scanning electron microscopy, fluorescence microscopy, and μ-Raman spectroscopy, are also used [162,163]. Pyr-GC/MS has recently been introduced for atmospheric MP analysis [164]. Models are currently being developed to estimate fluxes of atmospheric MPs’ emission, their transport, and their deposition. Such simulated data will contribute significantly to our understanding of the distribution of MPs on a global scale [161,165]. Future research should focus on developing standardized techniques and methods specific to atmospheric MPs. In addition, the atmospheric transport of MPs needs to be understood so that potential sources can be identified and mapped.

Airborne MPs can be ingested and inhaled by humans, thus allowing MPs to enter the digestive and respiratory systems. Inhalation of MPs, especially through indoor air, contributes to higher human exposure to MPs compared to other routes of exposure and, thus, directly poses a potential health risk [166].

Atmospheric pollution by MPs must be monitored over the long term and considered globally. It is necessary to compare the characteristics of atmospheric MPs with those of MPs from other environmental compartments. Moreover, the influence of MPs’ shape and size on transport and deposition in terrestrial or aquatic environments, even in remote areas, is also an important factor. Airborne MPs could adversely affect plants, animals, and humans. Therefore, there is an urgent need to work on mitigation measures to reduce the release and exposure to MPs.

## 7. Recommendations

The chemical composition of air, soil, sediment, and water is heterogeneous, resulting in a wide range of characteristics, functioning, and interactions with contaminants.

With all the methods described in this review, a comprehensive recovery of all the spiking MPs across all size classes was not attainable by one single method, and it is now evident that the type of matrix should be prioritized in deciding the extraction procedure, as each environmental compartment has its own complexity.

Each isolation technique has its own advantages and disadvantages, and ongoing attempts are being made to enhance existing methods and develop new ones that may increase the throughput, detection limit, and repeatability. When selecting an analytical technique, there are four primary criteria to consider: (i) examine the whole technique; (ii) evaluate the major merit of the various accessible methods; (iii) adapt methodology to channels and instruments; and (iv) construct integrated strategies and provide statistics on MPs. The ideal option is to test and employ an intelligent mix of data acquired from various approaches.

To ensure accurate results, procedures for each matrix must be carefully conducted to prevent cross-contamination. Environmental samples are susceptible to contamination by plastic particles from external sources if proper precautions are not taken during sampling and analysis. Failing to account for cross-contamination during the analysis and interpretation of the results can lead to a significant overestimation of MP quantities. Considering that MP pollution originates from plastic items, the obvious solution is to minimize their presence to reduce the risk of cross-contamination. When eliminating such items is not feasible, special cautionary measures and procedures should be developed to minimize the potential migration of pollutants. Various methods for mitigating cross-contamination exist, many of which are applicable universally, regardless of the sample type. Popular approaches include wearing clothing devoid of plastic fibers, thorough cleaning of laboratory surfaces and equipment, covering samples and laboratory apparatus, working under controlled airflow conditions, and using glass or metal laboratory dishes exclusively. Obtaining reliable results necessitates the implementation of relevant QA/QC measures. One such crucial step involves measuring cross-contamination through the analysis of control samples, including blanks [167,168,169].

**Figure 3:**
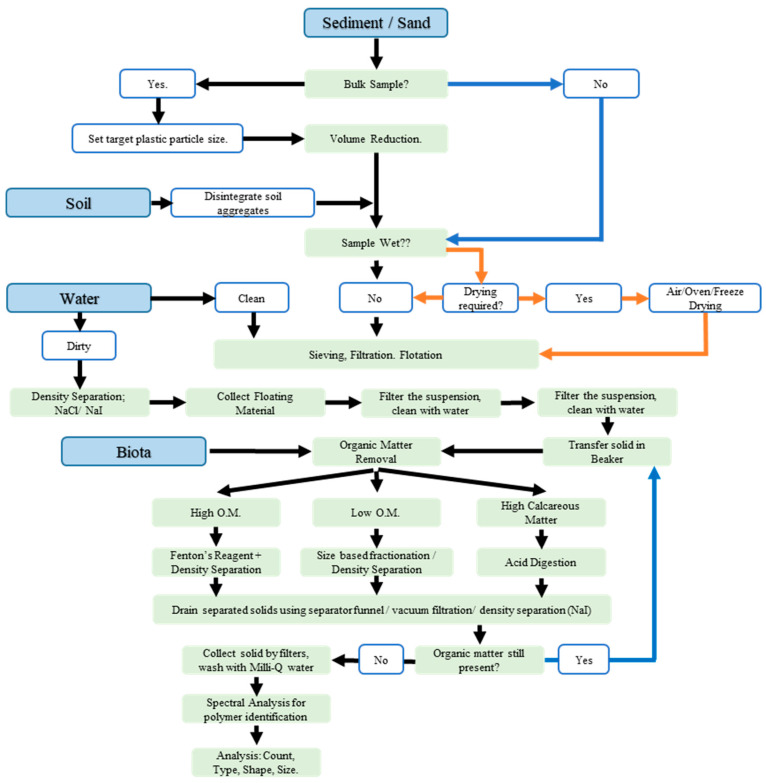
A roadmap for extraction of microplastics with respect to different environmental compartments.

It is imperative to take replicates of water, sediment, soil, and biota from different geographical landscapes and environments to increase the comparability and repeatability even among the same kind of matrix. Digestion modules, which frequently require case-by-case customization, are an excellent illustration of methodological heterogeneity dependent on sample type. A combination of different protocols should be tried and tested, particularly to extract MPs from organic-rich samples, such as wastewater, for example. Figure 3 presents a summary of extraction methods by sample type.

Moreover, a technical data sheet can be a powerful tool that allows the author to build a database of all the research that is going on in the MP community. Furthermore, in these technology-driven times, an application based on this sheet is highly recommended.

## 8. Future Research

The search of the analytical literature presented here reveals a proliferation of methods, which is a sign of the discipline’s health and expansion but can also have disastrous consequences for those who are seeking a method to solve a specific problem. It must be highlighted that substantial progress has been achieved in MP research in terms of analysis, interactions with other pollutants, toxicological effects, and removal by various treatment methods; nevertheless, there are still numerous knowledge gaps. The focus on MPs as a novel contaminant has resulted in a considerable rise in worldwide funding in this emerging study sector during the last decade, as can be estimated from the number of publications in this sector. MP research has come a long way in a short span of time, with no prior standardization in sampling, extraction, and identification methods. This review highlights the diversity in the extraction methods of MPs from all environmental compartments. This clearly resulted in a non-gold-standard approach that can be followed to reach the desired outcome. A set of guidelines should be laid in terms of matrix and location combined, and it should include time, size, quantity, and other factors expressed here. Before designing the method, the following key points should be given utmost importance and consideration:

- Goal of the study;
- Scientific hypothesis;
- Experimental factors: dependent and independent variables, repeatability, and reproducibility;
- Time efficiency;
- Cost of the procedure and reagents;
- Environmental health and safety: toxicity of reagents;
- Sample size;
- Large-scale applicability;
- Long-term goal;
- Qualitative and quantitative aspects.

It is recommended that raw data be shared among researchers on a common platform, ticking all the boxes laid by Cowger et al. [170] to assure that the findings can be replicated, as MP research is highly variable across space and time. It could possibly lead us one step closer to finding methods that are scalable and time- and cost-efficient. Furthermore, it is also advised that proof-of-concept studies be encouraged as a strategic framework to help environmental and analytical researchers to make decisions about future research and monitoring to develop better models to address the implications of MP research.

According to researchers, by 2050, a century after commercial plastic manufacturing began, the total amount of plastics produced will have topped 25 billion tons. The demand for plastics in low- and lower-middle-income countries is one-fifth that of their wealthier counterparts. In other words, established economies create more rubbish than emerging economies, closing the gap between countries’ net contributions to the problem of plastic pollution [171]. To put it another way, in the next 35 years, we will make and sell twice as much plastic as we did in the previous 65 years. Considering these parameters and the difficulties related to the study of MPs and a realization that plastic accumulation in ecosystems will eventually have an influence on our environment, it is necessary to build a sustainable relationship for the use of plastic based on global international collaboration.

## 9. Conclusions

In this review, we provided a comprehensive description of the methods used for extracting MPs based on different types of environmental matrices. Although the bulk of the techniques are utilized in comparable ways in many types of matrices, it is crucial to note that even minor changes in the approach can have a significant impact on the outcomes. The general need for improved environmental analytical methods has resulted in the launch of a new generation of extremely sensitive analytical equipment, as well as the development of novel MP extraction procedures.

Given the complexities of soil, sediment, and wastewater matrices, all the steps that lead to MP analysis are quite arduous. Many alternative methodologies have been developed to answer various concerns regarding MP contamination, including origins, transportation, and environmental destiny, as well as human and wildlife consequences. Despite the fact that certain technologies are still in their early stages, we can observe that automation is continually expanding the breadth of such devices’ uses in environmental studies in academic and commercial contexts. Although sample (matrix) processing is essential in practically all analytical methods, it is often overlooked as a significant phase in analytical chemistry, with major attention focused on the determination step. This idea of priorities is all too visible in the equipment and investment plans of many analytical laboratories. However, a positive trend in recent years indicates a greater understanding of the fundamental relevance of matrix processing in the pursuit of high-quality analytical results and valid conclusions.

## Figures and Tables

**Table 1:** Microplastic-extraction abilities of different hypersaline solutions. The + and − signs indicate the adequate (+) or inadequate (−) technique for that type of microplastics. Reference numbers are given in brackets. Typical densities of different polymers are reported in the first row. Prices refer to Merck website (update July 2023).

Salt	Density g/cm^3^	PET	HD PE	PVC	LD PE	PP	PS	PA	Remarks	Price EUR/kg
		1.32–1.41	0.94–0.96	1.14–1.46	0.91–0.92	0.85–0.92	1.04–1.08	1.12–1.15		
Milli-Q water	1	−	−	−	+	+	−	−	1. Easy to use 2. Low recovery rate	32.20
NaCl	1.2	−	+	±	+	+	+	+	1. Easy to use, non-toxic 2. Low recovery rate, requires multiple washings	46.50
ZnCl_2_	1.5–1.8	+	+	+	+	+	+ [46]	+	1. Reusable 2. Corrosive, strong foaming with organic samples [47,48] 3. Toxic to aquatic life [49]	139.00
NaI	1.55–1.8	+	+	±	+	+	+	+	1. Reusable 2. Reacts with cellulose fibers, hygroscopic, multistep method 3. Eye irritant	396
Sodium Polytungstate	1.4–1.65	+	+	+	+	+	+	−	1. Eye irritant 2. Toxic to aquatic life	266/100 g
Sodium Dihydrogen Phosphate monohydrate	1.4–1.45	+	+	+	+	+	+	−	1. Hazard free 2. Heating is required to achieve desired density [50]	96.60
CaCl_2_	1.3–1.35	+	+	+	+	+	+ [51]	−	1.Organic matter settles slowly due to high viscosity [32] 2. Ca^2+^ caused flocculation of organic substances through ion bridging; thus, it is not suggested for organic rich samples [48] 3. Eye irritant	97.60
ZnBr_2_ dihydrate	1.7	+	+	+	+	+	+	+	1. Toxic to aquatic life, eye irritant	344.00
NaBr	1.37	+	+	+	+	+	+	+ [52]	1. Eye irritant	80.60
Lithium tungstate	1.62	+	+	+	+	+	+	+	1. Recommended by NOAA	79.90/25 g
Potassium iodide	1.7	+	−	+	−	+	−	+	1. Eye irritant 2. Toxic to aquatic life [53]	298.00
K(HCOO)	1.5	+	+	+	+	+	+	+	1. Reusable	96/L
NaCl/NaI	1.2/1.8	+	+	+	+	+	+	+	1. High recovery rate 2. Pretreatment required [54]	−

**Table 2:** Extraction of microplastics based on elutriation technique.

Height	Width	Sieve Size	Optimal Conditions	Sample Amount	Saline Solution	Removal Efficiency	Reference
147 cm	15 cm	Top: 1 mm Bottom: the 35 μm mesh has the function of a sample holder, supported on a 1 mm mesh.	flux of 300 L/h of water for 15 min.	500 mL	NaI	93–98%	[69]
50 cm	5.06–10.16 cm	Top: 3 mm	385 L/h and 5.06 cm in column width for 10 min	500 mL	-	50%	[70]
186 cm	106 mm	Top: 63 and 32 μm	1.2 × 10^−2^ m/s and 1.9 × 10^−2^ m/s, for 300 s	50.5 g	-	92%	[71]

**Table 3:** Summary of oils used for extracting microplastics.

Oil Type	Viscosity	Matrix	Separator	Amount of Oil	Sample Mass	Extraction Time	Polymer Type	Mean Recovery Rate
Canola oil	86 cP	Aquatic sediment	Separatory funnel		50 g	90 to 168 min per sample	EPS, PVC, ABS, PA, and PES	96.1% ± 7.4
Canola oil	86 cP	Fluvial/artificial sediments (environmental samples)	Sediment microplastic isolator		10 g	15 min for water 45 min for sediment	EPS, CoPA, PA6, PE, PP, PS, PVC, PVDC, PET, and synthetic rubber	85.8%
Castor oil	580 cP	Marine beach sediments Agricultural soil Marine suspended surface solids Fluvial suspended surface solids	Separatory funnel		-	-	PP, PS, PMMA, and PET-G spiked samples	99% ± 4 95% ± 4%
Olive oil	84 cP	Soil Compost	Cylinder		25 g 10 g	-	PE, PS, PVC, PC, PET, and PU	90% ± 2% to 97% ± 5%
NaCl + olive oil	-	Sediment Water	Custom glassware with peristaltic pump		-	-	PP, PE, PA, EPS, and PET	82%
Mineral oil	95–100 cp	Sea sand Agricultural soil Sea sediment	-	0.25 g	2 g	10 s	PP, PS, LDPE, HPDE, PET, PVC, and PTFE	99%

**Table 5:** Organic-removal methods used in various studies.

S. No.	Matrix	Method	Main Composition	Temp	Digestion Time	Observations	Recovery	Ref.
1.	Organic-rich freshwater	Density separation with ZnCl_2_ + centrifugation	7% NaClO	50 °C	6 h–12 h	Nylon: digestion LDPE: centrifugation	94%	[89]
2.	Mussels	Oil extraction with NaCl	30% H_2_O_2_	60 °C	40 h	Study needed for small MPs Only performed for PP, PVC, and PET	95%	[126]
3.	Vegetal rich clayey sediment	Density separation with ZnCl_2_	30% H_2_O_2_	70 °C 100 °C 100 °C	1 h 3 h 7 h	Multiple digestion steps High temperature can degrade MPs		[127]
4.	Algae, driftwood, and feathers		H_2_O_2_+Fe^2+^	50 °C	1 h	Good for plant/organic-matter removal Cellulose acetate is degraded	65.9%	[128]
5.	Fish and muscles		KOH	50 °C	1 h	Good for animal tissues Cellulose acetate is degraded LDPE mass loss	58.3%	[128]
6.	Edible salt	Density separation with NaI + centrifugation	H_2_O_2_	65 °C 30 °C	24 h 48 h	MPs lost during centrifugation	95%	[129]

**Table 6:** Chemical resistance data of common plastics. R, resistant to degradation from chemical; PR, partially resistant; NR, not resistant.

S. No.	Reagent	%	PA	PC	PE	PET	PP	PS	PVC
1	HNO_3_	20	-	R	R	R	PR	R	R
2	HNO_3_ @ 50 °C	70	NR	NR	NR	NR	NR	NR	NR
3	HCl	35–36	NR	NR	R	NR	R	PR	R
4	HCl @ 50 °C	35–36	NR	NR	R	NR	R	PR	PR
5	H_2_SO_4_	30	-	R	R	R	R	R	R
6	H_2_SO_4_	95–98	NR	NR	PR	NR	PR	NR	PR
7	H_2_SO_4_ @ 50 °C	95–98	NR	NR	NR	NR	NR	NR	NR
8	KOH	30	R	NR	R	NR	R	R	R
9	KOH	50	R	NR	R	NR	R	R	R
10	KOH @ 50 °C	50	PR	NR	R	NR	R	R	PR
11	NaClO	30	NR	R	R	R	R	R	R
12	NaOH	30	R	NR	R	NR	R	R	R
13	NaOH	50	R	NR	R	NR	R	R	R
14	NaOH @ 50 °C	50	-	NR	R	NR	R	R	R
15	H_2_O_2_	30	NR	R	R	NR	R	R	R
16	H_2_O_2_	90	NR	R	RR	R	R	R	NR
17	H_2_O_2_ @ 50 °C	90	NR	R	R	-	PR	R	NR

**Table 7:** Cost of reagents used in organic-matter removal. Prices refer to Merck website (update July 2023).

S. No.	Reagent	Cost
1	HNO_3_	EUR 28.20/L
2	HCl 1M	EUR 29.80/L
3	H_2_SO_4_	EUR 41.00/L
4	KOH	EUR 44.80/kg
5	NaClO	EUR 23.72/L
6	NaOH	EUR 45.60/kg
7	H_2_O_2_	EUR 38.70/L
8	Protease A	EUR 147.00/mg
	Lipase FE-01	EUR 181/mg
	Amylase TXL	EUR 95.70/mg
	Cellulase TXL	EUR 36.00/g

## Data Availability

The data presented in this study are available upon request from the corresponding author.

## Abbreviations

ABS	acrylonitrile butadiene styrene
CaCl_2_	calcium chloride
CoPA	copolymer of Nylon 6 and Nylon 6/6
DCM	dichloromethane
DNA	Deoxyribonucleic acid
DW	dry weight
EPS	expanded polystyrene
EtOH	ethanol
FA	fly ash
Fe-NPs	iron nanoparticles
FTIR	Fourier-transform infrared
GHS	Globally Harmonized System of Classification and Labelling of Chemicals
HCl	hydrochloric acid
HClO_4_	perchloric acid
HDPE	high-density polyethylene
HNO_3_	nitric acid
H_2_O_2_	hydrogen peroxide
H_3_PO_4_	phosphoric acid
H₂SO_4_	sulfuric acid
ICES	International Council for the Exploration of the Sea
ISO	International Organization for Standardization
K(HCOO)	potassium formate
KOH	potassium hydroxide
KWS	Korona–Walzen–Scheider
LDPE	low-density polyethylene
MPs	Microplastics
MPSS	Munich plastic sediment separator
NaBr	sodium bromide
NaCl	sodium chloride
NaClO	sodium hypochlorite
NaI	sodium iodide
NaOH	sodium hydroxide
Na_2_WO_4_∙2H_2_O	sodium tungstate dihydrate
NOAA	National Oceanic and Atmospheric Administration
OEP	oil-extraction protocol
PA	polyamide
PC	polycarbonate
PCCPs	personal care and cosmetic products
PDMS	polydimethylsiloxane
PES	polyethersulfone
PET	polyethylene terephthalate
PFE	pressurized fluid extraction
PMMA	poly(methyl methacrylate)
POM	polyoxymethylene
PP	polypropylene
PS	polystyrene
PTFE	polytetrafluoroethylene
PU	polyurethane
PVC	polyvinyl chloride
Pyr-GC-MS	pyrolysis–gas chromatography–mass spectrometry
QA/QC	quality assurance and quality control
RIC	resin identification number
THF	tetrahydrofuran
UNEP	United Nations Environmental Programme
USEPA	US Environmental Protection Agency
UV	ultraviolet
WWTPs	wastewater treatment plants
ZnBr_2_	zinc bromide
ZnCl_2_	zinc chloride
